# Detection and quantification of groundnut oil adulteration with machine learning using a comparative approach with NIRS and UV–VIS

**DOI:** 10.1038/s41598-024-70297-7

**Published:** 2024-09-09

**Authors:** John-Lewis Zinia Zaukuu, Manal Napari Adam, Abena Amoakoa Nkansah, Eric Tetteh Mensah

**Affiliations:** Department of Food Science and Technology, Kwame Nkrumah University of Science and Technology, Kumasi, Ghana

**Keywords:** Near infrared, Ultraviolet–visible, Chemometrics, Adulteration, Free fatty acid, Peroxide value, Iodine value, Infrared spectroscopy, Near-infrared spectroscopy

## Abstract

Groundnut oil is known as a good source of essential fatty acids which are significant in the physiological development of the human body. It has a distinctive fragrant making it ideal for cooking which contribute to its demand on the market. However, some groundnut oil producers have been suspected to produce groundnut oil by blending it with cheaper oils especially palm olein at different concentrations or by adding groundnut flavor to palm olein. Over the years, there have been several methods to detect adulteration in oils which are time-consuming and expensive. Near infrared (NIR) and ultraviolet–visible (UV–Vis) spectroscopies are cheap and rapid methods for oil adulteration. This present study aimed to apply NIR and UV–Vis in combination with chemometrics to develop models for prediction and quantification of groundnut oil adulteration. Using principal component analysis (PCA) scores, pure and prepared adulterated samples showed overlapping showing similarities between them. Linear discriminant analysis (LDA) models developed from NIR and UV–Vis gave an average cross-validation accuracy of 92.61% and 62.14% respectively for pure groundnut oil and adulterated samples with palm olein at 0, 1, 3, 5, 10, 20, 30, 40 and 50% v/v. With partial least squares regression free fatty acid, color parameters, peroxide and iodine values could be predicted with R^2^CV’s up to 0.8799 and RMSECV’s lower than 3 ml/100 ml for NIR spectra and R^2^CV’s up to 0.81 and RMSECV’s lower than 4 ml/100 ml for UV–Vis spectra. NIR spectra produced better models as compared to UV–Vis spectra.

## Introduction

Food adulteration has long been a persistent problem in the food industry. The quality of food prepared for consumers is largely dependent on the quality of the component used in the making of these productsClick or tap here to enter text^1^. Edible oils are particularly prone to adulteration, whereby they are sometimes mixed with products of similar characteristics and of inferior quality. This deceptive act is mostly committed by retailers and occasionally by small scale manufacturers, primarily to increase their profits. Edible vegetable oils have always been a good source of essential fatty acids which is crucial in the physiological development of the human body^2^. Adulterating these oils can significantly reduce these beneficial benefits, leading to potential harm or threat to the health of consumers^3^. Edible oil adulteration typically encompasses two methods, it can either be done by blending of cold-pressed oil with refined oil, the substitution of more expensive oils with the cheaper alternatives^1^.

Among vegetable oils, groundnut oil is known for its rich oleic and linoleic content, with excellent oxidative stability which makes it an ideal cooking and frying)^4^. The oil is also recognized for its ability to reduce the risk of cardiovascular diseases. It is characterized by a strong pleasant aroma which contributes to its demand on the market which makes it highly appreciated and sells at a very good price. However, these inherent characteristics make groundnut oil susceptible to adulteration. One prevalent adulteration method involves blending groundnut oil with palm olein oil in varying proportions. A more deceptive method involves production of fake groundnut oil by incorporating groundnut flavor into cheaper oils)^2^, specifically palm olein.

Palm olein is a physically clear liquid at 25 ℃ obtained from palm fruit. It contains about 45% and 35% oleic and linoleic acids respectively and an iodine value < 60. It can be further fractionated into super palm olein and palm top olein. Further fractionation results in higher iodine value (> 60). Despite the differences in iodine values, the oleins are all suitable for cooking, hence the general name “palm olein”^5^.

There have been several methods for detecting adulteration in oil over the years. Some of these methods include, chemical testing, sensory analysis and instrumental testing)^2^. Spectroscopy is one of the most commonly used instrumental methods, primarily chosen because of its rapid screening capabilities and non-destructive nature^1^. Ultraviolet visible (UV–Vis) and near infrared (NIR) spectroscopy are considered cheap and fast spectroscopic methods for analysis^6^. NIR spectroscopy uses electromagnetic radiation ranging from 780 to 2500 nm, when combined with chemometrics also serves as a valuable tool in food monitoring^7^. The technique has been applied for the detection of flour in yoghurt^8^, detection of heat treated honey^9^, characterization probiotic supplements^10^ and the detection of coriander oil adulteration^11^. UV–Vis coupled with chemometrics is often used to detect adulteration in various food products with oil being a prominent example. For instance, a recent study by Gao et al.^12^, applied this technique in detecting adulteration of Extra Virgin Olive Oil.

Notably, in Ghana, the economic discrepancy between palm olein and groundnut oil, primarily due to the ease of cultivation of the oil palm fruit compared to other oil crops, its abundant yields, and its ability to bear fruit all year round^13^ consequently makes it an ideal adulterant for groundnut oil.

The study focuses on a novel approach to rapidly detect and quantify groundnut oil adulteration using NIR and UV–Vis spectroscopy in combination with advanced chemometric techniques. Unlike previous studies that focused on different forms of adulteration and also, on a single spectroscopic method or limited chemometric analysis, this study offers a comprehensive comparison of two spectroscopic methods, providing a deeper understanding of their respective capabilities and limitations in detecting groundnut oil adulteration. In addition, there has no reported study for the detection of palm olein in groundnut oil. By developing and validating robust chemometric models, the study will compare NIR spectroscopy and UV–Vis in terms of accuracy and predictive power for key quality parameters such as free fatty acids, iodine value, and peroxide value. This dual-method approach not only enhances the reliability of adulteration detection but also offers a cost-effective and rapid alternative to traditional, more labor-intensive methods. Thus, this study significantly advances the field of food adulteration detection, offering practical solutions for quality control in the food industry.

## Materials and methods

### Sample acquisition

Authentic groundnut oil was purchased from a reliable source from the Northern region of Ghana. This oil was also used in the preparation of the laboratory sample (adulterated oil) and the development of calibration models.

#### Sample preparation

Pure groundnut oil was adulterated with palm olein at nine different concentrations (Table 1) to mimic suspected adulterations of those on the market. Each concentration was prepared in triplicates using a pipette, resulting in 27 laboratory repared samples in total. Every varying concentration added up to a total of 100 mL and was stored in transparent plastic containers at room temperature.

**Table 1:** Ratio of mixing for sample preparation.

Sample code	Palm olein conc (% v/v)	Groundnut oil conc (% v/v)
GP00	0	100
GP01	1	99
GP03	3	97
GP05	5	95
GP10	10	90
GP20	20	80
GP30	30	70
GP40	40	60
GP50	50	50

In addition to the laboratory samples, groundnut oil samples were obtained from three different major markets in the Northern region, which were referred to as market 1 to 3 in this study. Fifteen ground oil samples were purchased from each of the three markets resulting in a total of 45 market samples. These market samples were used for validation of the developed models and were not included in the calibration set. The laboratory adulterated samples together with the pure samples (27 samples) and market samples (45 samples) were scanned with the UV–VIS and NIR spectrophotometers.

#### Near infrared scanning (NIR)

The NIR spectra were obtained using NIR-S-G1 (InnoSpectra Co., Hsinchu, Taiwan), a handheld spectrophotometer with wavelength ranging from 900 to 1700 nm. The spectra were collected in transmittance mode with a spectral resolution of 3 nm. Each sample was recorded three consecutive times, giving a total of 216 spectra (72 samples × 3 consecutive scans). All samples were scanned at room temperature in Ziploc bags.

#### UV–Vis scanning

A UV–Vis spectrophotometer (Mettler Toledo GmbH, Switzerland) equipped with a plastic cuvette with 10 mm path length was used. Spectra were collected at 1 nm interval between a wavelength of 200 nm to 1100 nm at room temperature. The samples comprised of 9 laboratory prepared samples which were prepared in triplicates as well as samples bought from 5 different sellers from 3 different markets, which were also done in triplicates, Making a total of 72 samples. Each sample was recorded three consecutive times for each replicate, adding up to a total of 216 spectra. The instrument was blanked on deionised water.

### Physicochemical parameters

Free fatty acids, peroxide value, saponification, colour and iodine value were the parameters analysed. All parameters were measured in triplicates for all samples analyzed using standard methods.

#### Determination of free fatty acids

The free fatty acid was determined for all samples according to the^14^ method Approximately 2.0 g of oil were used, dissolved in 25 mL of ether-alcohol solution (2:1), as well as two drops of 1% phenolphthalein indicator in ethanol. Titration was performed using 0.1 M sodium hydroxide solution in deionized water until the appearance of pink color, with a minimum persistence of 30 s. The % Free Fatty Acid for each sample was calculated using the formula below.

$$\frac{ml of alkali used\times N-alkali}{weight of sample}\times M$$

The means and standard deviations were then calculated and recorded. FFA was calculated as %oleic acid.

#### Determination of peroxide value

Peroxide value was determined according to the Cd 8-53 method of the American Oil Chemists' Society (AOCS), with minor modifications. Two grams (2 g) of the groundnut oil sample was weighed in a conical flask, 30 mL of Glacial Acetic acid/ Chloroform solution (3:2) was added to the groundnut oil sample, and the solution was swirled until it dissolved. 0.50 ml of saturated KI solution was added and allowed to stand for 1 min with occasional shaking. 30 ml of distilled water was added and titrated with 0.1 M Na_2_SO_3_ using 0.5 mL starch as an indicator. Blank titration was performed without oil samples. The peroxide value was expressed as mEq/kg.

#### Determination of iodine value

The Wijs method according to^15^ was used to determine the iodine value. A mixture of 1 g of groundnut oil sample and 2 mL chloroform was added into 5 mL of Wijs solution, the conical flask was closed with a ground glass stopper, swirled and allowed to stand for 5 min in the dark. 3 mL of a 7.5% aqueous KI was added to the solution. The final content was titrated with 0.1 N sodium thiosulphate (Na_2_SO_3_) solutions using 0.1 µL starch as an indicator, and blank was conducted in the same manner as the sample but without oil.

#### Determination of color

Using a pipette, 30 mL of oil samples were measured into petri dishes. The color parameters were measured with a Chroma meter (Konica Minolta brand Chroma meter), model CR_400 with illuminate D65 and 2° angle of observation. The parameters were measured using the CEILAB (L*a*b*) color system. Where L* ranging from 0 to 100 (0 = black, 100 = colorless) measured the clarity of the samples, a* ranging from − 60 to + 60 (− 60 = green, + 60 = red) measured the redness/green color component of the samples and b* also ranging from − 60 to + 60 (− 60 = blue, + 60 = yellow) measured the yellow/blue color component of the sample^16^.

### Multivariate data analysis

Spectroscopic analysis was carried out in R Studio software. Savitzky-Golay smoothing preprocessing method was applied to the raw spectra obtained from both spectrophotometers before developing classification models.

#### Principal component analysis

Principal component analysis (PCA) was used for data visualization. The use of Principal Component Analysis (PCA) serves to reduce the dimensions of spectral data, providing insights into the potential for distinguishing objects, while also generating Principal Component (PC) scores. PCA is an unsupervised method that condenses the significant variations within a spectral dataset into the initial principal components, which maintain orthogonality among themselves. Importantly, PCA is applied without regard to the correlation between the dependent and independent variables. It employs linear pattern recognition techniques to identify and remove outliers. In this study, we initially conducted PCA analysis on the spectra of each sample type. Subsequently, we created a three-dimensional scatter plot of scores for the first three principal components (PCs) to visualize how effectively the nine categories of ingredient samples could be differentiated.

#### Parial least square-discriminant analysis (PLS-DA)

PLSDA was used to classify market samples and adulterated laboratory samples and classify different concentrations of the adulterant (palm olein) in the market samples. In this study, PLS-DA was used to design and optimized classification models using principal component scores. PLS-DA was initially developed to classify the authentic samples and various types of adulterants, irrespective of their concentrations as well as the market samples. Subsequently, the models was used to distinguish between different concentration levels using first the complete dataset, and after using specific datasets corresponding to the adulterants, with concentration as the class variable. To assess the predictive accuracy of these PLS-Da models, the dataset was split into training (calibration) and test (validation) sets. Lastly, PLS-DA models were developed for the classification of the market samples in comparison with pure groundnut oil. For all developed models, leave-one-sample-out cross validation was used. Each model was validated using a leave-one-sample-out validation (LOSO), where all scans of one sample concentration were left out in each iteration step of the cross-validation. The statistical parameters used to evaluate the performance of the PCA-LDA models were the recognition accuracy (%) and prediction accuracy (%). Recognition accuracy (%), represents the accuracy of calibration, whereas prediction accuracy (%), represents the accuracy of cross-validation (%). These were assessed through confusion matrices where columns represented the actual class membership and the rows represented the predicted class membership. Other parameters used to evaluate the performance of the developed PCA-LDA models were the sensitivity, specificity and precision^17^ calculated after cross-validation as followed:

$$Sensitivity \, = \, True \, positives/\left( {True \, positives \, + False \, negative} \right)$$

$$Specificity \, = \, True \, negative/\left( {True \, negative \, + \, False \, negative} \right)$$

$$Precision \, = \, True \, positives/\left( {True \, positives \, + \, False \, positives} \right)$$

#### Partial least square regression

Partial least square regression (PLSR) was used to quantitatively predict the iodine value, free fatty acid content and peroxide value present in the laboratory samples irrespective of the adulterant concentration. PLSR was also used in the prediction of adulterant concentration and the total color change in the laboratory samples. 18 different pretreatments were applied to get the most accurate PLSR model^18^. Leave-one-sample-out Cross Validation was employed to test the predictive significance of the models. All spectra from single sample were excluded during model training at a time. These are the excluded spectra to evaluate the models predictive accuracy. This was repeated for all samples, effectively utilizing the available data for training and validation. The model performances were assessed according to the values of Root Mean Square Error of Calibration (RMSE), Coefficient of Determination (R^2^) and for Cross-Validation, RMSECV and R^2^CV.The optimum number of latent variables was determined based on the minimum RMSECV value to minimize the probability of over fitting. The “aquap2” package in R-project was used for all spectral evaluations.

### Statistical analysis of conventional analytical procedures

#### Evaluation of physicochemical parameters

The data was analyzed using Excel and Stat tools package in Palisade statistical software. Mean and standard deviation were calculated for the triplicate measures. Analysis of variance (ANOVA) was used to determine significant differences among samples. The analysis was done at a confidence interval of 95%.

## Results and discussion

### Physicochemical properties of laboratory adulterated samples

**Figure 1:**
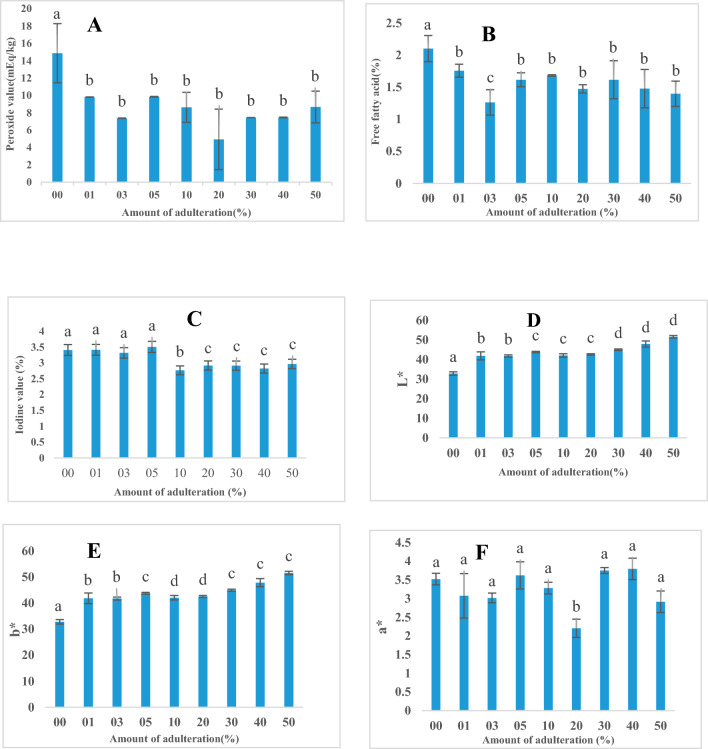
Average values of physical and chemical properties of pure and laboratory adulterated samples. (**A**) Peroxide value. (**B**) Free fatty acid. (**C**) Iodine value. (**D**) Color coordinate L*. (**E**) Color coordinate b*. (**F**) Color coordinate a* (p < 0.05). Different alphabets represent samples with significant differences.

Average results for physical and chemical analysis of prepared adulterated samples are shown in Fig. 1. Peroxide value is frequently used to calculate the total hydroperoxide content and monitor the rate of lipid oxidation during refining and preservation of oil^19^. It displays the amount of peroxides measured in milliequivalents (mEq) of active oxygen per kilogram of oil^20^. Iodine value is a parameter which expresses the degree of unsaturation in oils which reflects the susceptibility of the oil to oxidation^21^. Free fatty acids are produced by hydrolysis of oils during oxidation making them prone to oxidation and rancidity^22^. From Fig. 1A–C, it can be seen that the pure sample (0%) was significantly different from the other samples and recorded a high peroxide value (14.86 ± 3.41 meq/kg) and free fatty acid (2.10 ± 0.20) as compared to the other samples. According to studies done by^23^, it was observed that groundnut oil and palm olein blends had decreasing values of free fatty acid, peroxide and iodine values with increasing concentrations of palm olein. Similar observations can be seen in the prepared laboratory adulterated samples. Palm olein has lesser free fatty acid due to the rigorous refining process it undergoes. This explains the decrease in free acid content of the oil blends. The decrease in iodine value could be due to the fact that increasing concentrations of palm olein decreased the levels of unsaturation characterized by groundnut oil. Lastly, decreased levels of peroxide value could be attributed to a decrease in peroxide concentration of the oil blends.

The color parameters of the samples were examined based on L*, a* and b* color coordinates. The L* value shows brightness, positive a* value shows redness, negative a* shows greenness, positive b* value shows yellowness and negative b* value shows blueness. From D (Fig. 1), the lighter sample (L*) was the pure sample (32.83 ± 0.86) while the darkest (least bright) was the sample with 50% adulteration (51.62 ± 0.65). From E (Fig. 1), the most yellow sample (+ b*)was the sample with 50% adulteration (32.22 ± 0.62). and the least yellow sample was the pure sample with a b* value of 7.08 ± 0.86. From F, the reddest sample (+ a*) observed was the sample with 40% adulteration (3.79 ± 0.28) and the least red sample (2.20 ± 0.24) was the sample with 20% adulteration. Overall, the colour analysis showed that all the oil blends recorded lightness within acceptable levels which is the preference of consumers.

### Raw and pre-treated spectra analysis for NIR and UV–Vis

**Figure 2:**
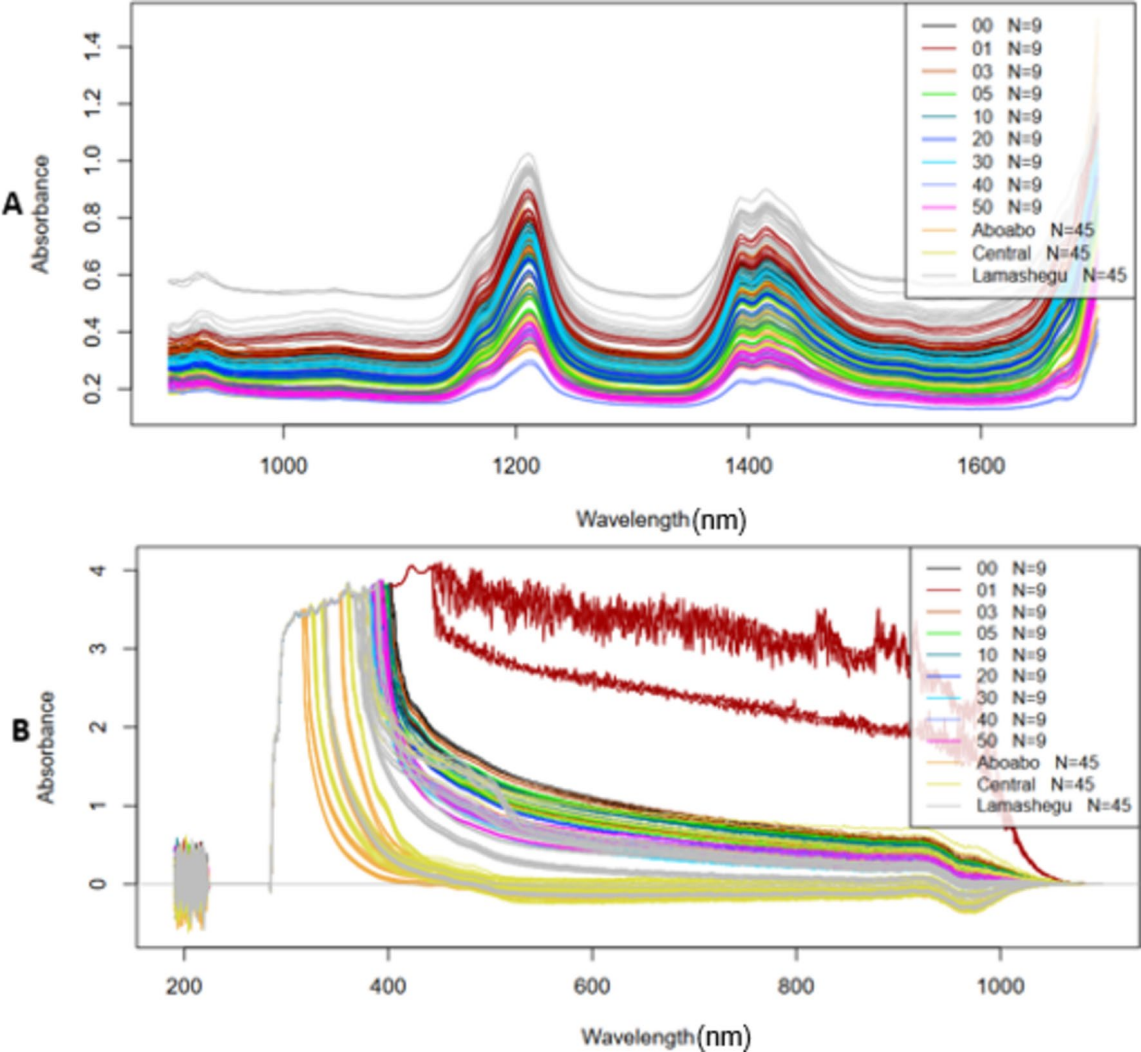
Raw spectra of laboratory adulteration and market samples (**A**) NIR raw spectra. (**B**) UV–VIS raw spectra.

**Figure 3:**
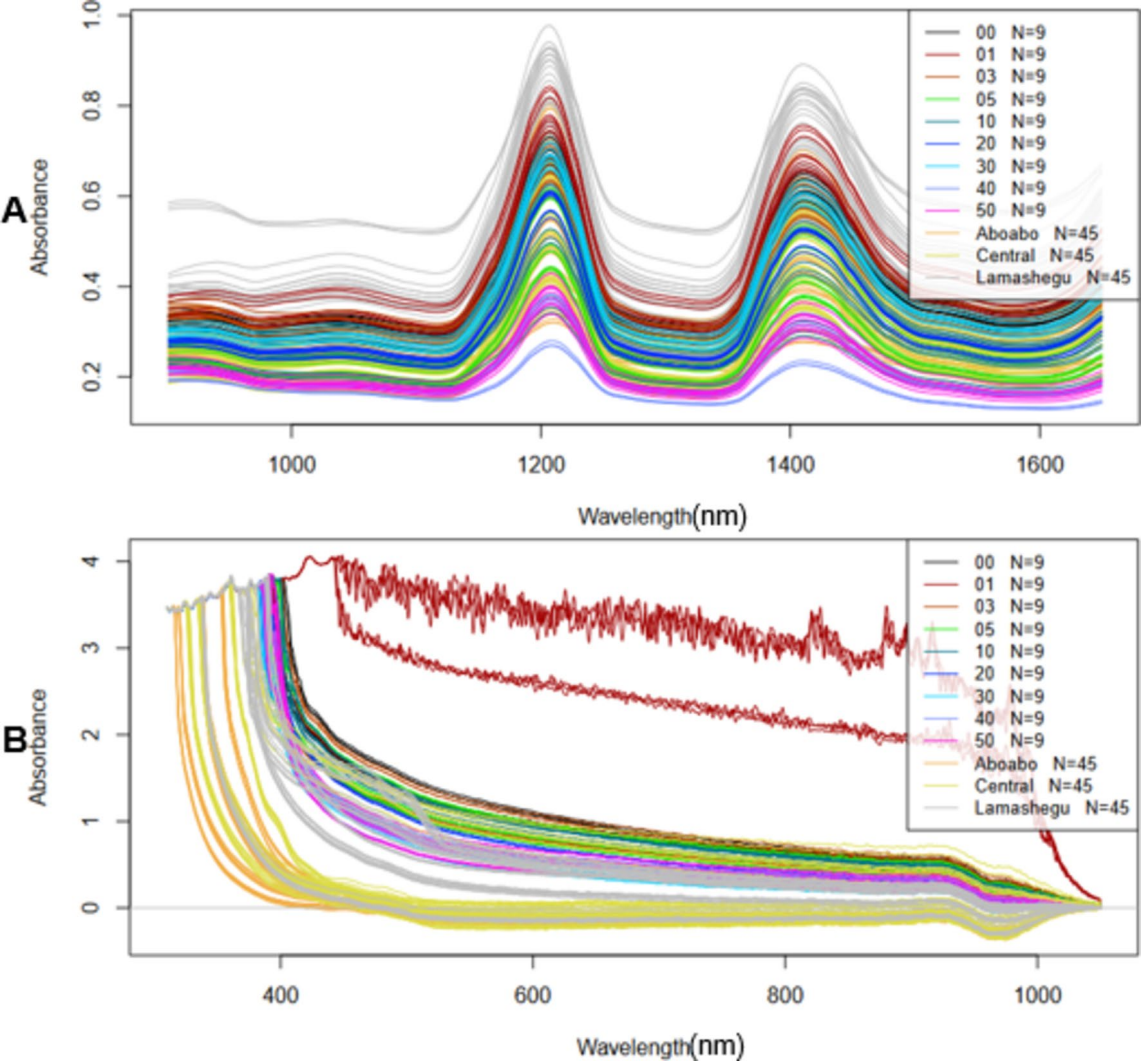
Preprocessed spectra of laboratory adulteration and market samples (**A**) NIR preprocessed spectra. (**B**) UV–VIS preprocessed spectra.

Figure 2 shows the raw spectra of laboratory adulteration and market samples using NIR spectroscopy (A) and UV–Vis (B). raw spectra. Figure 3 shows the pretreated (sgol) spectra of laboratory adulteration and market samples using NIR spectroscopy (A) and UV–Vis (B).

From the raw spectra of the NIR spectroscopy analysis (Fig. 2A), the laboratory adulterated samples had absorbances up to 0.8 and the market samples had absorbances up to 1.0 from the NIR raw spectra. Several key peaks can be observed which correspond to various chemical bonds and functional groups in the samples. NIR spectroscopy typically detects overtones and combinations of fundamental molecular vibrations, which are usually associated with C–H, O–H, and N–H bonds. Peaks around 1200–1500 nm can be attributed to the second overtone of C–H stretching vibrations^24^. These are indicative of the aliphatic hydrocarbon chains present in oils. Peaks observed between 1600 and 1800 nm are likely due to combination bands involving C–H stretching and bending vibrations. This region can provide insights into the overall hydrocarbon content. Although less prominent, any peaks around 1400–1450 nm may suggest the presence of moisture or hydroxyl groups^25^. The peaks were still visible after preprocessing (Fig. 3A).

From the raw spectra of the UV–Vis analysis (Fig. 2B), the laboratory adulterated samples had absorbances up to 3.5 and market samples had absorbances up to 4.0 for UV–Vis raw spectra. In the Ultraviolet–Visible (UV–Vis) preprocessed spectra, distinct absorption peaks are observed which can be linked to various chromophores and conjugated systems within the samples. Peaks in the UV region (200–400 nm) are generally due to electronic transitions in aromatic compounds or conjugated dienes. These peaks are crucial for identifying the presence of specific unsaturated compounds or antioxidants within the oils^26^. Peaks around 400–500 nm can indicate the presence of non-bonding electrons transitioning to anti-bonding π orbitals. These transitions are typical in compounds with lone pairs such as carbonyls. Peaks beyond 500 nm into the visible range can be attributed to the color compounds in the oils, such as carotenoids and chlorophylls^27^.The peaks were still visible after preprocessing (Fig. 3B).

### PCA scores for NIR and UV–VIS spectra

**Figure 4:**
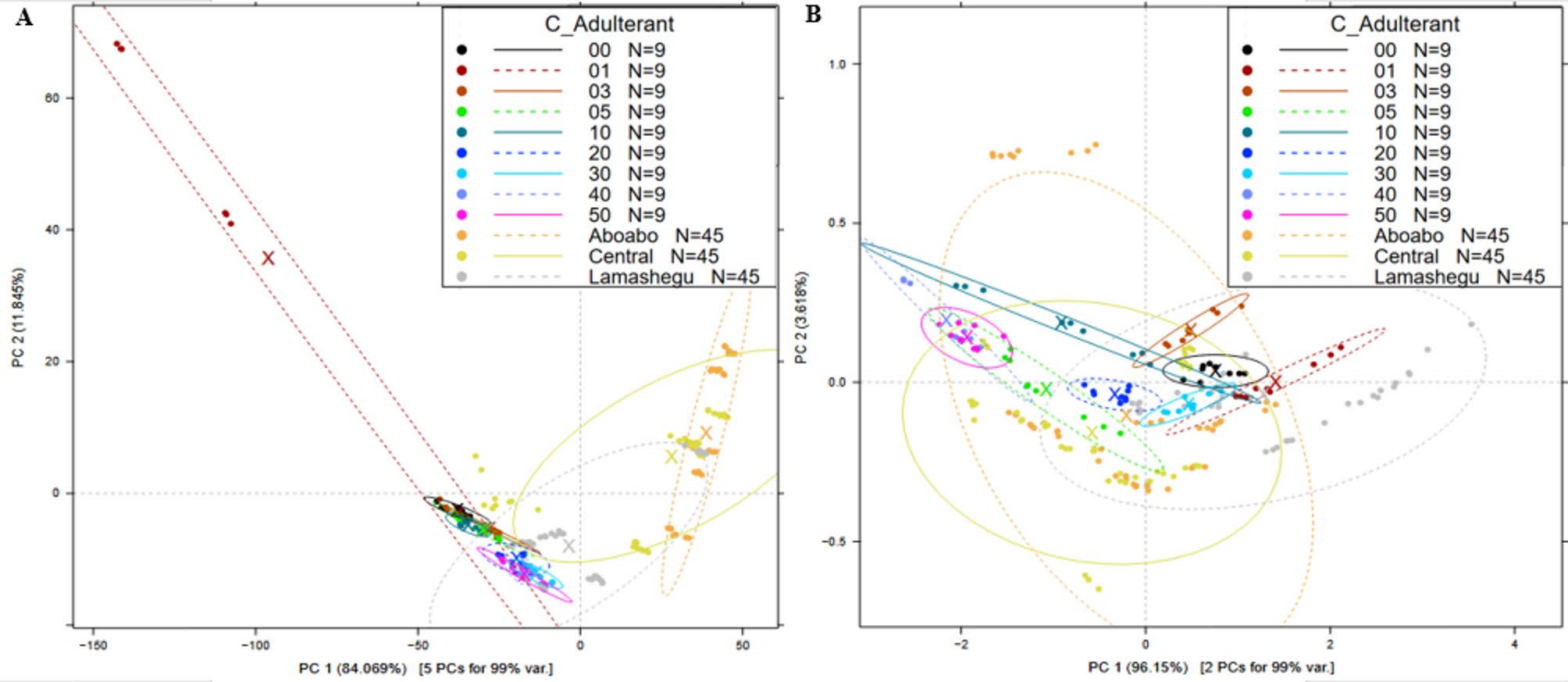
PCA plot for laboratory adulterated laboratory and market samples (**A–C**) UV–Vis PCA plot for laboratory adulterated laboratory and market samples spectra (**B**). NIR PCA plot for laboratory adulterated laboratory and market samples.

In order to group comparable samples closer together, PCA, an unsupervised pattern recognition technique, extracts important information. This results in the visualization of data trends in a three-dimensional space as shown in Fig. 4. In this sense, determining the differences between the various sample categories used might be done using the graphical output^28^. Based on PCA analysis of pretreated spectra of NIR spectra of differently adulterated and market samples, the first two principal components (PC1 and PC2) described a total variance of 99.76% (Fig. 4A). From the PCA score plot, samples from Abaobo market showed similarities with samples from market Central. Samples from Lamashegu market also showed similarities with samples with 0%, 1%, 20% and 30% of adulterant. Which indicates that the NIR spectra have distinct differences that can be effectively used to differentiate between the samples. The close grouping of samples from Abaobo and Central markets, as well as the overlap of Lamashegu market samples with adulterated ones, suggest similar chemical compositions and potential adulteration. For Near-Infrared (NIR) spectra, wavelengths associated with overtones and combinations of molecular vibrations such as C–H, O–H, and N–H stretching are important. These wavelengths contain information about the oil's fatty acid composition, moisture content, and other minor constituents that affect the oil's quality and stability.The absorption bands around 1200–1500 nm are significant due to their sensitivity to changes in fatty acid profiles, which directly relate to properties like free fatty acid content and iodine value^29^. Also, regions around 1700–1900 nm are indicative of moisture and other volatiles in the oil, influencing its peroxide value and overall oxidative stability. In the UV–Visible (UV–VIS) spectra, wavelengths beyond 500 nm are particularly critical as they correspond to the visible range where color compounds such as carotenoids and chlorophylls absorb. These compounds are indicators of the oil's purity and quality, with specific peaks indicating the presence and concentration of these pigments.Wavelengths around 450–550 nm are associated with the yellow and green color components, which can differentiate between pure groundnut oil and adulterated samples due to their varying pigment contents. The clustering observed in the PCA plots reflects these differences, with distinct groupings based on the spectral signatures related to the oil's physicochemical properties like color, peroxide value, and fatty acid.

It can then be said that samples from Lamashegu market could have some form of adulteration as shown in the resemblance they have with the adulterated laboratory samples in the PCA score plot. For the pretreated spectra of UV–VIS, the first principal component PC1 showed a variance of 84.06% and PC2 showed a variance of 11.84%. From Fig. 4B, laboratory adulterated samples (0 to 50%) were in the negative variance in PC1 signifying similarities between them and samples from Lameshegu market. This suggests that UV–Vis spectra also effectively differentiate the samples but with less variance explained compared to NIR. The similarities in PC1 for laboratory adulterated samples and Lameshegu market samples reinforce the potential adulteration observed in these samples. Scree plot and PCA loadings for both UV–Vis and NIR dataset has been provided in the supplementary document (Figs. S1 and S2).

### LDA models for NIR and UV–VIS spectra

#### LDA for NIR

**Figure 5:**
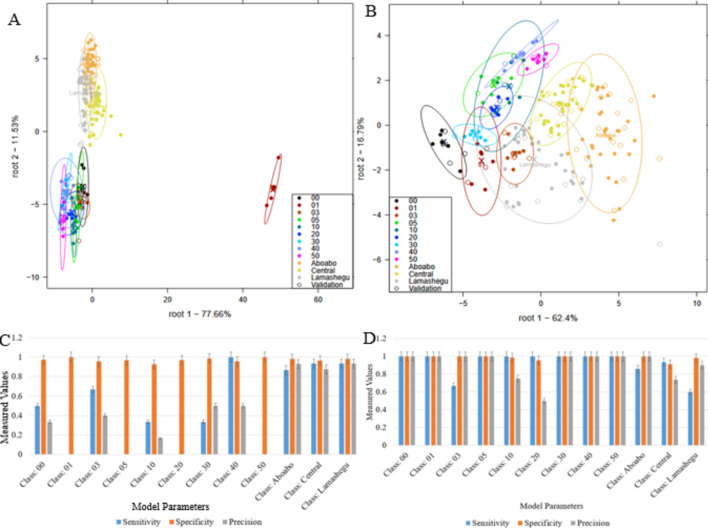
LDA plot for laboratory adulterated and market samples (**A**–**C**) LDA and model parameters for UV–Vis (**B**–**D**) LDA and Model Parameters for NIRS.

Classification plots and model performance parameters for the detection of groundnut oil adulteration can be seen in Fig. 5.The results of the LDA models built from the NIR spectra to classify the laboratory adulterated and market samples are shown in Fig. 5A. It was observed that there was a degree of overlap among all laboratory samples suggesting that they all contained groundnut oil and palm olein. Samples from Aboabo and Central markets appeared to be different from the laboratory adulterated samples but showed some overlapping with each other and with samples from Lamashegu market. Furthermore, samples from Lamashegu market showed overlapping with laboratory adulterated samples at 1%, 3%, 5%, 10%, 20% and 30% w/wconcentrations. For Fig. 5C, High sensitivity for laboratory-adulterated samples (close to 100% for 0%, 1%, 20%, 30%, 40%, and 50% adulteration). High specificity for these samples, as they can be accurately classified without significant overlap. Precision is also high, with most samples being correctly identified without false positives.

#### LDA for UV–Vis

In LDA model for the UV–VIS spectra, the laboratory adulterated samples showed a significant degree of overlap except for sample containing 1% adulteration as seen in Fig. 5B. In addition, all market samples showed overlapping patterns with each other but did not extend to the laboratory as seen in the LDA model for the NIR spectra. For Fig. 5D, Lower sensitivity for distinguishing between different levels of adulteration due to significant overlap among samples. Lower specificity as the model struggles to differentiate between similar samples. Lower precision due to higher misclassification rates. Recall and F1 values can be found in Tables S1 and S2 in the supplementary sheet. For the NIR spectra, the significant wavelengths are those that can effectively distinguish between laboratory adulterated samples and market samples. This includes the wavelengths where there is significant absorption due to the presence of groundnut oil and palm olein as depicted in the raw spectra. plots. For the UV–VIS spectra, although the LDA model showed significant overlap among samples.

#### Confusion matrices from NIR and UV–VIS spectra

Table 2 shows the confusion matrix for the classification of the laboratory adulterated and market samples. There was an average recognition of 95.09% and average prediction of 92.61%. The confusion matrix shows their accuracies of classifications and misclassifications. Laboratory adulterated samples with 0%, 1%, 20%, 30%, 40% and 50% concentrations could be classified with 100% accuracy. Higher levels of misclassification were observed for samples 3%, 5%, 10% and all market samples meaning they share similarities with each other. It is likely that they all contain groundnut oil and palm olein but in varying concentrations.

**Table 2:** Confusion matrix developed from NIR spectra for laboratory adulterated and market samples.

		00	01	03	05	10	20	30	40	50	Aboabo	Central	Lamashegu
Average prediction accuracy: 92.61% Average recognition accuracy: 95.09%	00	100	0	0	0	0	0	0	0	0	0	0	0
	01	0	100	0	0	0	0	0	0	0	0	0	2.2
	03	0	0	89	0	0	0	0	0	0	0	0	0
	05	0	0	0	89	0	0	0	0	0	0	0	0
	10	0	0	0	11	77.93	0	0	0	0	0	2.2	0
	20	0	0	0	0	11.04	100	0	0	0	0	0	8.87
	30	0	0	0	0	0	0	100	0	0	0	0	0
	40	0	0	0	0	11.04	0	0	100	0	0	0	0
	50	0	0	0	0	0	0	0	0	100	0	0	0
	Aboabo	0	0	0	0	0	0	0	0	0	93.18	2.2	6.67
	Central	0	0	0	0	0	0	0	0	0	6.82	95.6	15.54
	Lamashegu	0	0	11	0	0	0	0	0	0	0	0	66.71

Table 3 presents the confusion matrix for the classification of both laboratory and market samples for the UV–VIS spectra. It shows an average prediction accuracy of 62.17% and an average recognition accuracy of 96.13%. Higher levels of misclassification were observed for all samples therefore making the LDA model built from the NIR spectra a better one as it showed better accuracy and less misclassification.

**Table 3:** Confusion matrix for the classification of the laboratory and market samples for the UV–VIS spectra.

		00	01	03	05	10	20	30	40	50	Aboabo	Central	Lamashegu
Average prediction accuracy: 62.14% Average recognition accuracy: 96.13%	00	50	0	0	0	0	0	0	0	0	0	4.46	0
	01	25.19	0	22.33	0	0	0	0	0	0	0	0	0
	03	0	11	55.67	0	0	0	0	11.04	0	0	0	0
	05	12.41	22.33	0	66.67	22.33	0	0	0	0	0	0	0
	10	0	11	11	33.33	77.67	0	0	0	0	0	0	0
	20	0	0	11	0	0	22.26	0	11.04	22.33	0	0	0
	30	0	0	0	0	0	33.22	66.67	0	11	0	0	0
	40	0	0	0	0	0	22.26	33.33	66.89	0	0	0	0
	50	0	0	0	0	0	22.26	0	11.04	66.67	0	0	0
	Aboabo	12.41	0	0	0	0	0	0	0	0	88.87	0	6.67
	Central	0	33.33	0	0	0	0	0	0	0	11.13	91.07	0
	Lamashegu	0	22.33	0	0	0	0	0	0	0	0	4.46	93.33

### Partial least square regression for NIRS

#### Prediction of free fatty acid, iodine, peroxide value and colour

**Figure 6:**
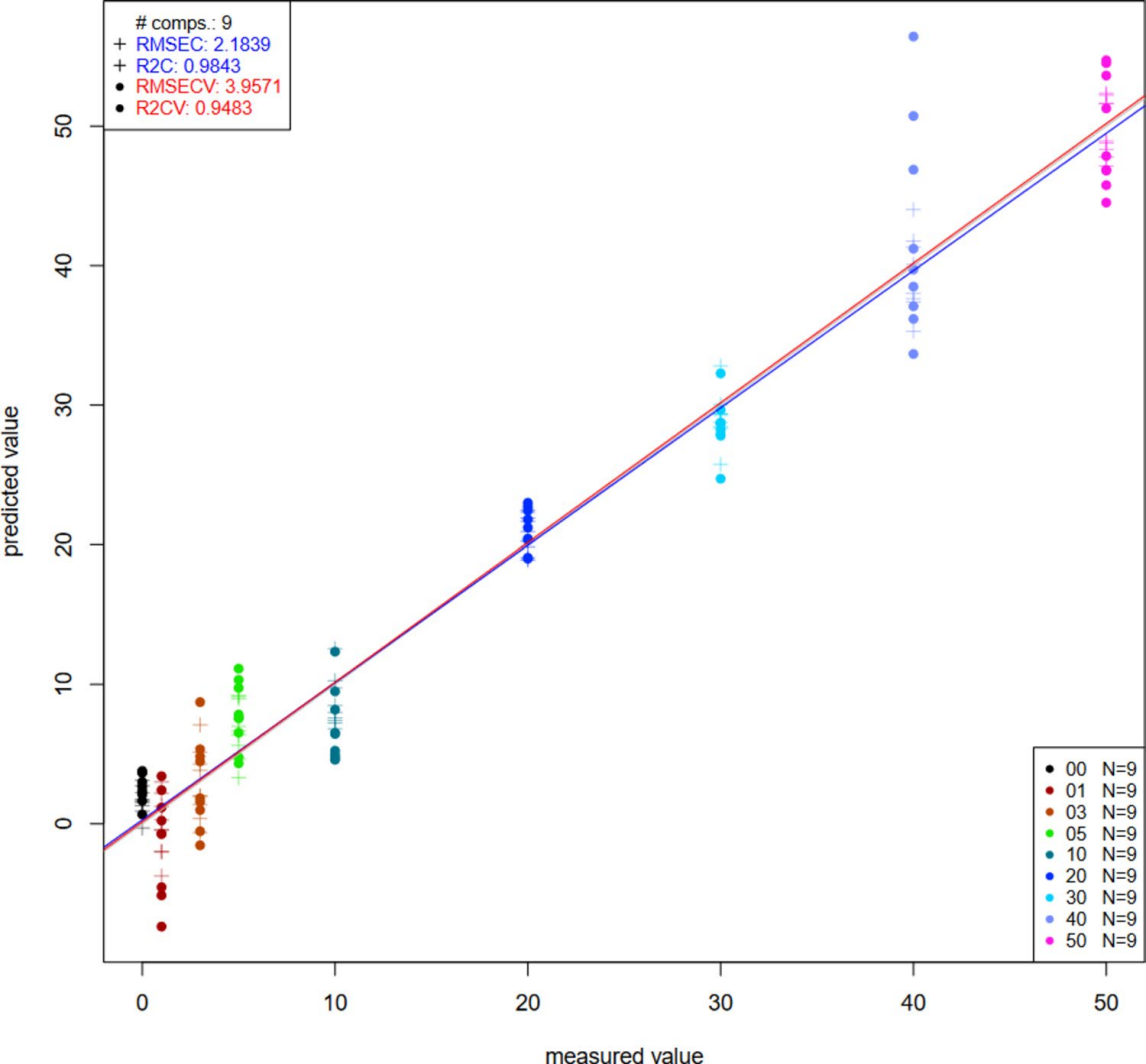
PLSR plot for the prediction of palm olein concentration on groundnut oil using NIRS using Savitzky-Golay Pretreatment (filter 17).

After 18 different pretreatments were applied (Tables S3 to S6, supplementary sheet), R^2^ values ranged from 0.7735 to 0.94 while R^2^CV values ranged from 0.4712 to 0.7847. After cross validation, the errors (RMSECV) developed were all lower than 1 mL/100 mL for fatty acid prediction (Table 4). Savitzky-Golay smoothing (sgol) with filter 17 pretreatment produced the best accuracy for the prediction of palm olein in groundnut oil using NIRS (Fig. 6).

**Table 4:** Partial least square values obtained from Savitzky-Golay pretreatment applied to the NIR spectra for the prediction of free fatty acid, iodine, peroxide values and color.

	R^2^	RMSE (mL/100 mL)	R^2^CV	RMSECV (mL/100 mL)
Concentration	0.98	2.18	0.94	3.95
Iodine value	0.8775	0.09	0.65	0.16
Free fatty acid	0.8973	0.07	0.784	0.10
Peroxide value	0.7691	1.23	0.51	1.78
L*	0.9434	1.14	0.87	1.68
a*	0.727	0.24	0.30	0.39
b*	0.9439	1.60	0.88	2.33

For iodine values prediction (Table 4), it was observed that R^2^ value was 0.8775, while R^2^CV value was0.656. Again, the errors (RMSECV) after cross validation were below 1 mL/100 mL. Savitzky-Golay smoothing (sgol) with filter 17 pretreatment proved to have the best accuracy for predicting iodine value in groundnut oil regardless of the adulterant concentration.

In the case of peroxide prediction (Table 4), R^2^ value was 0.7691, while R^2^CV had avalue of 0.5169. The errors developed after cross validation (RMSECV) were all lower than 3 mL/100 mL. Savitzky-Golay smoothing (sgol) with filter 17 then detrending (deTr) produced the best accuracy for predicting peroxide value in groundnut oil irrespective of the adulterant concentration.

The coefficient of determination (R^2^), ranging between 0 and 1, is one of the indicators of a model’s quality. If the value is higher or nearer 1, it acts as a better model. If it is farther away from one, it indicates a poor model^30^. The root mean square (RMSE) measures the average prediction error made by the model in predicting the outcome for an observation. The lower the RMSE, the better the model since it indicates the stability of the models. Conversely, higher values for RMSE show many errors hence a bad model. The preferred models were chosen based on the pretreatment that gave the highest R^2^ value and the lowest RMSE values. The best models which produced the highest R^2^ and R^2^CV while producing the least RMSE and RMSEC values were obtained from the pretreatments that involved the use of Savitzky-Golay smoothing (sgol) with filter 17 and Savitzky-Golay smoothing (sgol) with filter 17 then detrending (deTr). These pretreatments resulted in R^2^ of 0.8973, R^2^CV of 0.7847, RMSE of 0.0731 and RMSECV of 0.1059 for free fatty acid prediction (Table 4), R^2^ of 0.8775, R^2^CV of 0.656, RMSE of 0.0961 and RMSECV of 0.1611 for iodine value prediction (Table 4) and R^2^ of 0.7691, R^2^CV of 0.5169, RMSE of 1.2348 and RMSECV of 1.7861 for peroxide value prediction (Table 4).

The PLS model for prediction of colour in groundnut oil adulteration, with pre-processing involving Savitzky-Golay smoothing, showed R^2^ of 0.9434, R^2^CV of 0.8799, RMSE of 1.1477 and RMSECV OF 1.686 for lightness, R^2^ of 0.727, R^2^CV of 0.3074, RMSE of 0.2494 and RMSECV of 0.3971 for redness and R^2^ of 0.9439, R^2^CV of 0.8819, RMSE of 1.6093 and RMSECV of 2.3357 for yellowness. These results show a better model for colour prediction among differently adulterated samples since they had higher values of R^2^ while producing lesser values for RMSE.

### Partial least square regression for UV–Vis

**Figure 7:**
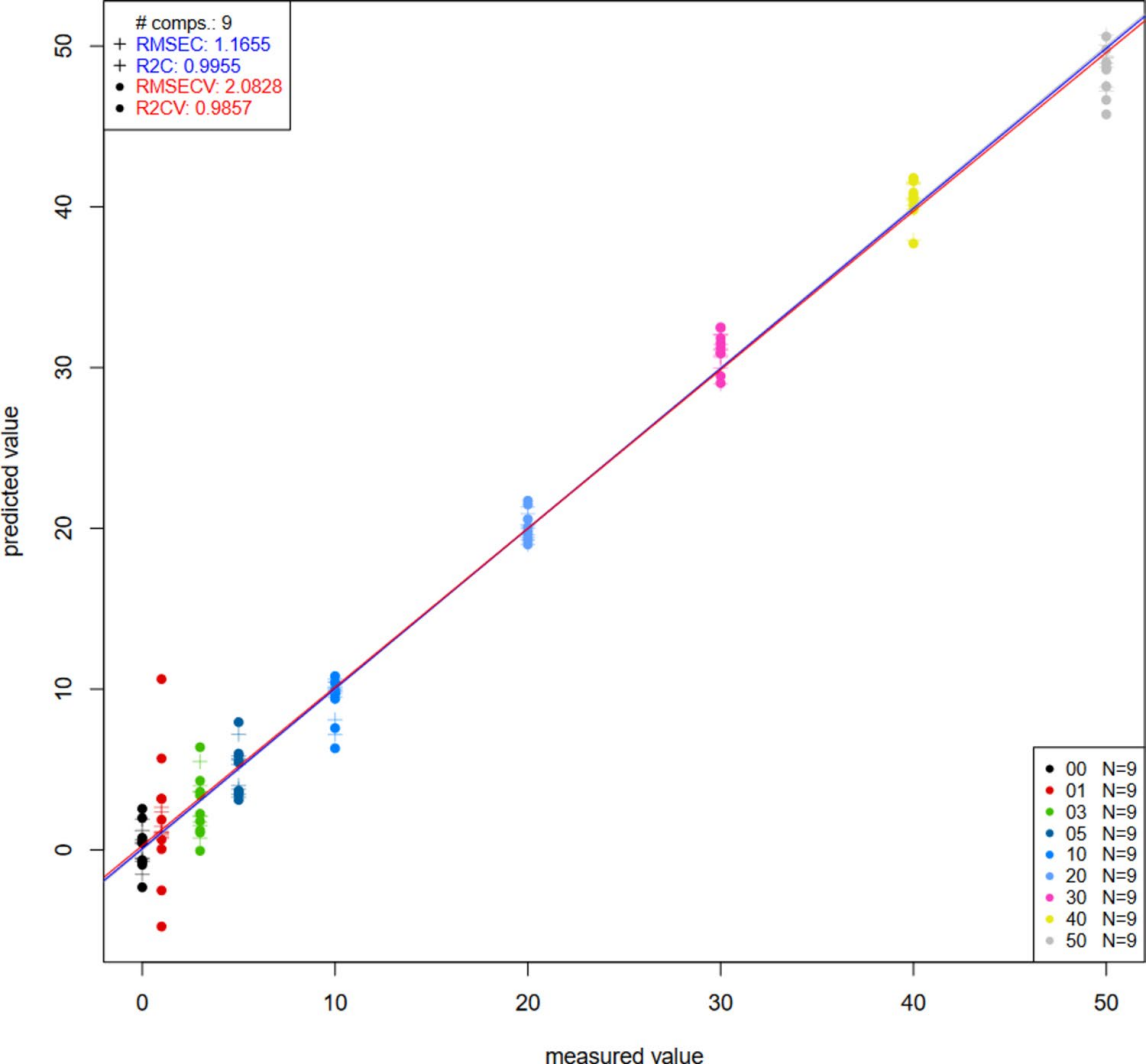
PLSR plot for the prediction of palm olein concentration on groundnut oil using UV–Vis using Savitzky-Golay Pretreatment (filter 17).

Overall, NIRS could predict all the parameters of interest better than UV–VIS (Table 5). Only the concentration of palm olein, brightness (L*) and yellowness (b*) of the samples could be predicted with UV–VIS. These were the same parameters that were also best predicted using NIRS but with higher accuracies. Models for all the other parameters were weak. Figure 7 shows the PLSR plot for the prediction of palm olein in groundnut oil (the best model achieved with UV–Vis).

**Table 5:** Partial least square values obtained from Savitzky-Golay pretreatment applied to the UV–VIS spectra for the prediction of free fatty acid, iodine, peroxide values and color.

	R^2^	RMSE (mL/100 mL)	R^2^CV	RMSECV (mL/100 mL)
Concentration	0.99	1.16	0.98	2.08
Iodine value	0.5013	0.19	0.43	0.2151
Free fatty acid	0.4844	0.16	0.25	0.1979
Peroxide value	0.6455	1.53	0.31	2.1273
L*	0.81	2.10	0.715	2.5758
a*	0.2906	0.40	− 0.0405	0.4868
b*	0.8001	3.04	0.7005	3.7189

The PLS models for UV–VIS pre-processed spectra for the prediction of free fatty acid, iodine, peroxide values and colour parameters in groundnut oil adulteration with palm olein were less satisfactory since they produced most R^2^ values farther from 1 and larger RMSE values as compared to the PLSR models from the NIR pre-processed spectra.

### Limitations

Some limitations of this study included the small study area and sample sizes used. Future studies could explore wider study areas to produce a more comprehensive understanding.

## Conclusion

The primary objective of this study was to utilise Near Infrared spectroscopy and UV–Vis spectroscopy coupled with chemometrics to develop models for the prediction and quantification of offered suitable methods for the detection of adulteration of groundnut oil with palm olein. PCA scores revealed a degree of overlapping between laboratory adulterated samples and market samples, indicating similarities between them. The LDA models were used to distinguish between pure groundnut oil and adulterated samples of varying concentrations using the NIR and UV–Vis spectroscopy. The data constructed yielded average cross-validation values of 92.61% and 62.14% respectively.

Moreover, PLSR was used for the prediction of physicochemical parameters such as iodine value, peroxide value, free fatty acid content and colour of the laboratory samples. For NIR spectra, recorded R^2^CV value of up to 0.8799 and RMSECV values lower than 3 mL/100 mL whereas UV–Vis spectra recorded R^2^CV values up to 0.3869 and RMSECV values lower than 4 mL/100 mL. Notably, NIR spectroscopy out-performed UV–Vis in developing more accurate predictive models for the detection and quantification of adulteration in groundnut oil. Other chemometric algorithms could be also be explored in future studies with a higher sample size.

## Supplementary Information

Supplementary Information.

## Data Availability

Data presented in this study are available on request from the corresponding author.
