# Supplementary material for: Detection and quantification of groundnut oil adulteration with machine learning using a comparative approach with NIRS and UV–VIS
Source: Sci Rep. 2024 Sep 9;14:20931. doi: 10.1038/s41598-024-70297-7 (PMC11383961; doi:10.1038/s41598-024-70297-7)
Supplement: Supplementary file 1 — Supplementary Information. [file 41598_2024_70297_MOESM1_ESM.docx]

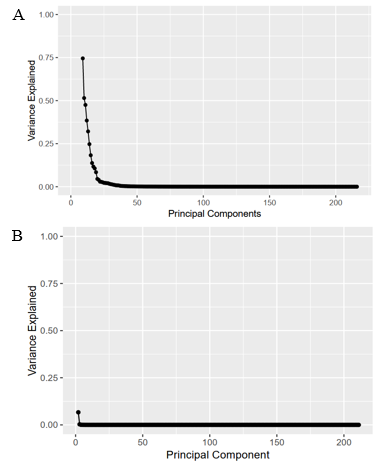


Figure S1: Scree plots for A. NIR and B. UV-Vis dataset


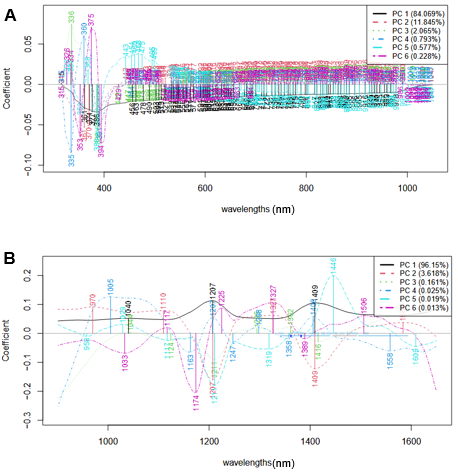


Figure S2: PCA loadings plots for **A.**  UV-Vis dataset and **B**. NIR dataset

Table S1: LDA model parameters using NIRS

|  | Sensitivity | Specificity | Precision | Recall | F1 |
| --- | --- | --- | --- | --- | --- |
| Class: 00 | 1 | 1 | 1 | 1 | 1 |
| Class: 01 | 1 | 1 | 1 | 1 | 1 |
| Class: 03 | 0.67 | 1 | 1 | 0.66 | 0.8 |
| Class: 05 | 1 | 1 | 1 | 1 | 1 |
| Class: 10 | 1 | 0.98 | 0.75 | 1 | 0.85 |
| Class: 20 | 1 | 0.95 | 0.5 | 1 | 0.66 |
| Class: 30 | 1 | 1 | 1 | 1 | 1 |
| Class: 40 | 1 | 1 | 1 | 1 | 1 |
| Class: 50 | 1 | 1 | 1 | 1 | 1 |
| Class: Aboabo | 0.85 | 1 | 1 | 0.85 | 0.92 |
| Class: Central | 0.93 | 0.91 | 0.73 | 0.93 | 0.82 |
| Class: Lamashegu | 0.6 | 0.98 | 0.9 | 0.6 | 0.72 |

Table S2: LDA model parameters using UV-VIS

|  | Sensitivity | Specificity | Precision | Recall | F1 |  |
| --- | --- | --- | --- | --- | --- | --- |
| Class: 00 | 0.5 | 0.97 | 0.33 | 0.5 | 0.4 |  |
| Class: 01 | 0.6 | 1 | 0.45 | 0.67 | 0.56 |  |
| Class: 03 | 0.66 | 0.95 | 0.4 | 0.66 | 0.5 |  |
| Class: 05 | 0.56 | 0.97 | 0.52 | 0.45 | 0.55 |  |
| Class: 10 | 0.33 | 0.92 | 0.16 | 0.33 | 0.22 |  |
| Class: 20 | 0.63 | 0.97 | 0.45 | 0.45 | 0.45 |  |
| Class: 30 | 0.33 | 0.98 | 0.5 | 0.33 | 0.4 |  |
| Class: 40 | 1 | 0.95 | 0.5 | 1 | 0.66 |  |
| Class: 50 | 0.72 | 1 | 0.53 | 0.52 | 0.47 |  |
| Class: Aboabo | 0.86 | 0.98 | 0.92 | 0.86 | 0.89 |  |
| Class: Central | 0.93 | 0.96 | 0.87 | 0.93 | 0.90 |  |
| Class: Lamashegu | 0.93 | 0.98 | 0.93 | 0.93 | 0.93 |  |

Table S3: Partial least square values obtained from 18 pretreatments applied to the NIRS spectra for the prediction of concentration

| pretreat | NrLV | R^2^ | RMSE (mL/100mL) | R^2^CV | RMSECV (mL/100mL) |
| --- | --- | --- | --- | --- | --- |
| **sgol@2-17-0** | **9** | **0.9843** | **2.1839** | **0.9483** | **3.9571** |
| sgol@2-19-0 | 9 | 0.9632 | 3.3371 | 0.9072 | 5.3015 |
| sgol@2-17-0_snv | 9 | 0.9663 | 3.1954 | 0.9144 | 5.0919 |
| sgol@2-19-0_snv | 9 | 0.9818 | 2.35 | 0.9391 | 4.2965 |
| sgol@2-17-0_msc | 9 | 0.9741 | 2.8015 | 0.8944 | 5.6543 |
| sgol@2-19-0_msc | 9 | 0.971 | 2.9632 | 0.8851 | 5.8984 |
| sgol@2-17-0_deTr | 9 | 0.9678 | 3.1226 | 0.9207 | 4.9015 |
| sgol@2-19-0_deTr | 9 | 0.9658 | 3.2208 | 0.9168 | 5.0203 |
| sgol@2-17-0_deTr_snv | 9 | 0.9591 | 3.5186 | 0.9059 | 5.3384 |
| sgol@2-19-0_deTr_snv | 9 | 0.9549 | 3.6969 | 0.8981 | 5.5548 |
| sgol@2-17-0_deTr_msc | 9 | 0.959 | 3.5231 | 0.9074 | 5.2972 |
| sgol@2-19-0_deTr_msc | 9 | 0.9547 | 3.7027 | 0.8998 | 5.5088 |
| sgol@2-19-0_sgol@2-19-1 | 9 | 0.9633 | 3.3352 | 0.9223 | 4.852 |
| sgol@2-19-0_sgol@2-19-2 | 9 | 0.9525 | 3.7942 | 0.9119 | 5.1648 |
| sgol@2-17-0_sgol@2-17-1 | 9 | 0.9651 | 3.2517 | 0.9261 | 4.7321 |
| sgol@2-17-0_sgol@2-17-2 | 9 | 0.9622 | 3.3822 | 0.9145 | 5.0892 |
| sgol@2-19-0_sgol@2-19-1_deTr | 9 | 0.9585 | 3.5438 | 0.917 | 5.0137 |
| sgol@2-17-0_sgol@2-17-1_deTr | 9 | 0.9601 | 3.4764 | 0.9218 | 4.8678 |

Table S4: Partial least square values obtained from 18 pretreatments applied to the NIR spectra for the prediction of Free Fatty Acid

| pretreat | NrLV | R^2^ | RMSE (mL/100mL) | R^2^CV | RMSECV (mL/100mL) |
| --- | --- | --- | --- | --- | --- |
| **sgol@2-17-0** | **9** | **0.8973** | **0.0731** | **0.7847** | **0.1059** |
| sgol@2-19-0 | 9 | 0.8956 | 0.0737 | 0.7832 | 0.1062 |
| sgol@2-17-0_snv | 8 | 0.7747 | 0.1083 | 0.4991 | 0.1615 |
| sgol@2-19-0_snv | 8 | 0.7735 | 0.1086 | 0.4998 | 0.1614 |
| sgol@2-17-0_msc | 8 | 0.7816 | 0.1066 | 0.5039 | 0.1607 |
| sgol@2-19-0_msc | 9 | 0.807 | 0.1002 | 0.503 | 0.1609 |
| sgol@2-17-0_deTr | 9 | 0.8969 | 0.0733 | 0.7671 | 0.1101 |
| sgol@2-19-0_deTr | 9 | 0.8955 | 0.0737 | 0.7775 | 0.1076 |
| sgol@2-17-0_deTr_snv | 9 | 0.8453 | 0.0898 | 0.6088 | 0.1427 |
| sgol@2-19-0_deTr_snv | 9 | 0.8451 | 0.0898 | 0.6177 | 0.1411 |
| sgol@2-17-0_deTr_msc | 9 | 0.8121 | 0.0989 | 0.5424 | 0.1544 |
| sgol@2-19-0_deTr_msc | 9 | 0.8149 | 0.0982 | 0.5519 | 0.1528 |
| sgol@2-19-0_sgol@2-19-1 | 9 | 0.8701 | 0.0823 | 0.7042 | 0.1241 |
| sgol@2-19-0_sgol@2-19-2 | 9 | 0.844 | 0.0901 | 0.5765 | 0.1485 |
| sgol@2-17-0_sgol@2-17-1 | 9 | 0.8615 | 0.0849 | 0.6797 | 0.1291 |
| sgol@2-17-0_sgol@2-17-2 | 9 | 0.8362 | 0.0923 | 0.4712 | 0.1659 |
| sgol@2-19-0_sgol@2-19-1_deTr | 9 | 0.859 | 0.0857 | 0.7067 | 0.1236 |
| sgol@2-17-0_sgol@2-17-1_deTr | 9 | 0.8667 | 0.0833 | 0.7092 | 0.123 |

Table S5: Partial least square values obtained from 18 pretreatments applied to the NIR spectra for the prediction of Iodine value

| pretreat | NrLV | R^2^ | RMSE (mL/100mL) | R^2^CV | RMSECV (mL/100mL) |
| --- | --- | --- | --- | --- | --- |
| **sgol@2-17-0** | **9** | **0.8775** | **0.0961** | **0.656** | **0.1611** |
| sgol@2-19-0 | 9 | 0.875 | 0.0971 | 0.6633 | 0.1594 |
| sgol@2-17-0_snv | 9 | 0.7883 | 0.1264 | 0.38 | 0.2163 |
| sgol@2-19-0_snv | 9 | 0.7837 | 0.1277 | 0.38 | 0.2163 |
| sgol@2-17-0_msc | 1 | 0.2486 | 0.2381 | 0.1535 | 0.2527 |
| sgol@2-19-0_msc | 1 | 0.2485 | 0.2381 | 0.1533 | 0.2527 |
| sgol@2-17-0_deTr | 7 | 0.801 | 0.1225 | 0.599 | 0.1739 |
| sgol@2-19-0_deTr | 7 | 0.7937 | 0.1248 | 0.5951 | 0.1748 |
| sgol@2-17-0_deTr_snv | 2 | 0.4333 | 0.2067 | 0.2068 | 0.2446 |
| sgol@2-19-0_deTr_snv | 2 | 0.4305 | 0.2073 | 0.2031 | 0.2452 |
| sgol@2-17-0_deTr_msc | 2 | 0.4321 | 0.207 | 0.2038 | 0.2451 |
| sgol@2-19-0_deTr_msc | 2 | 0.4293 | 0.2075 | 0.2001 | 0.2456 |
| sgol@2-19-0_sgol@2-19-1 | 9 | 0.8599 | 0.1028 | 0.6315 | 0.1667 |
| sgol@2-19-0_sgol@2-19-2 | 5 | 0.6271 | 0.1677 | 0.4617 | 0.2015 |
| sgol@2-17-0_sgol@2-17-1 | 8 | 0.8441 | 0.1085 | 0.6262 | 0.1679 |
| sgol@2-17-0_sgol@2-17-2 | 9 | 0.7658 | 0.1329 | 0.4289 | 0.2076 |
| sgol@2-19-0_sgol@2-19-1_deTr | 4 | 0.6282 | 0.1675 | 0.4887 | 0.1964 |
| sgol@2-17-0_sgol@2-17-1_deTr | 4 | 0.6203 | 0.1692 | 0.472 | 0.1996 |

Table S6: Partial least square values obtained from 18 pretreatments applied to the NIR spectra for the prediction of peroxide value

| pretreat | NrLV | R^2^ | RMSE (mL/100mL) | R^2^CV | RMSECV (mL/100mL) |
| --- | --- | --- | --- | --- | --- |
| **sgol@2-17-0** | **9** | **0.7691** | **1.2348** | **0.5169** | **1.7861** |
| sgol@2-19-0 | 9 | 0.7184 | 1.3638 | 0.4624 | 1.8843 |
| sgol@2-17-0_snv | 9 | 0.6473 | 1.5263 | 0.0171 | 2.5918 |
| sgol@2-19-0_snv | 9 | 0.6383 | 1.5455 | 0.0126 | 2.5535 |
| sgol@2-17-0_msc | 1 | 0.6126 | 1.5995 | 0.0695 | 2.4789 |
| sgol@2-19-0_msc | 1 | 0.6009 | 1.6235 | 0.0313 | 2.5293 |
| sgol@2-17-0_deTr | 7 | 0.7566 | 1.2677 | 0.4549 | 1.8973 |
| sgol@2-19-0_deTr | 7 | 0.7622 | 1.2532 | 0.5172 | 1.7856 |
| sgol@2-17-0_deTr_snv | 2 | 0.7629 | 1.2514 | 0.3611 | 2.054 |
| sgol@2-19-0_deTr_snv | 2 | 0.7283 | 1.3395 | 0.3141 | 2.1283 |
| sgol@2-17-0_deTr_msc | 2 | 0.707 | 1.3909 | 0.2074 | 2.2879 |
| sgol@2-19-0_deTr_msc | 2 | 0.6768 | 1.4611 | 0.173 | 2.337 |
| sgol@2-19-0_sgol@2-19-1 | 9 | 0.6962 | 1.4164 | 0.374 | 2.0332 |
| sgol@2-19-0_sgol@2-19-2 | 5 | 0.7146 | 1.373 | 0.3172 | 2.1235 |
| sgol@2-17-0_sgol@2-17-1 | 8 | 0.6925 | 1.425 | 0.3873 | 2.0115 |
| sgol@2-17-0_sgol@2-17-2 | 9 | 0.7243 | 1.3493 | 0.2292 | 2.2561 |
| sgol@2-19-0_sgol@2-19-1_deTr | 4 | 0.6973 | 1.4139 | 0.4589 | 1.8904 |
| sgol@2-17-0_sgol@2-17-1_deTr | 4 | 0.751 | 1.2822 | 0.4457 | 1.9133 |

Table S7: Partial least square values obtained from 18 pretreatments applied to the UV-VIS spectra for the prediction of concentration

| pretreat | NrLV | R^2^ | RMSE (mL/100mL) | R^2^CV | RMSECV (mL/100mL) |
| --- | --- | --- | --- | --- | --- |
| sgol@2-17-0 | 9 | 0.9955 | 1.1655 | 0.9857 | 2.0828 |
| sgol@2-19-0 | 9 | 0.9894 | 1.7894 | 0.8699 | 6.2766 |
| sgol@2-17-0_snv | 9 | 0.9929 | 1.4671 | 0.9621 | 3.3888 |
| sgol@2-19-0_snv | 9 | 0.993 | 1.4583 | 0.9623 | 3.3813 |
| sgol@2-17-0_msc | 9 | 0.9767 | 2.6585 | 0.2608 | 14.9632 |
| sgol@2-19-0_msc | 9 | 0.9767 | 2.6562 | 0.2659 | 14.9115 |
| sgol@2-17-0_deTr | 9 | 0.994 | 1.3537 | 0.8941 | 5.664 |
| sgol@2-19-0_deTr | 9 | 0.9939 | 1.3558 | 0.9029 | 5.4245 |
| sgol@2-17-0_deTr_snv | 9 | 0.9956 | 1.1607 | 0.9854 | 2.0998 |
| sgol@2-19-0_deTr_snv | 9 | 0.9895 | 1.7816 | 0.8595 | 6.5243 |
| sgol@2-17-0_deTr_msc | 9 | 0.9919 | 1.5665 | 0.773 | 8.2928 |
| sgol@2-19-0_deTr_msc | 9 | 0.992 | 1.5539 | 0.7929 | 7.9201 |
| sgol@2-19-0_sgol@2-19-1 | 9 | 0.9685 | 3.0883 | 0.2129 | 19.167 |
| sgol@2-19-0_sgol@2-19-2 | 9 | 0.8386 | 6.9919 | 0.0215 | 17.2158 |
| sgol@2-17-0_sgol@2-17-1 | 9 | 0.9671 | 3.1565 | 0.276 | 19.6591 |
| sgol@2-17-0_sgol@2-17-2 | 9 | 0.8283 | 7.2124 | 0.1356 | 18.5459 |
| sgol@2-19-0_sgol@2-19-1_deTr | 9 | 0.9677 | 3.1266 | 0.1249 | 18.4587 |
| sgol@2-17-0_sgol@2-17-1_deTr | 9 | 0.9662 | 3.1981 | 0.1733 | 18.8517 |
